# On the Temperature of Children in Health and Disease

**Published:** 1846-04

**Authors:** 


### De la Temperature chez les Enfans a l'etat Physiologique et Pathologique. Par le Docteur *Henri Roger.* Paris, 1844-5.

### On the Temperature of Children in Health and Disease. By *Henry Roger,* M.D.

Dr. Roger has just completed an interesting series of articles in the *Archives Generales de Medicine,* upon the variations of Temperature in Children; and the conclusions at which he has arrived (based upon about a thousand thermometrical experiments) seem to us to possess sufficient novelty and importance to justify our laying a complete analysis of the Essay before our readers.

Numerous as have been the researches undertaken in respect to the animal heat of the healthy body, little has been done for the investigation of the changes of temperature in its diseased condition; and yet the subject is of great interest, for while the mean temperature of man varies scarcely a degree at the poles and the equator, even a slight attack of illness may immediately produce a great thermometrical difference. It is true we have some isolated observations upon the subject, such as those of MM. Becquerel and Breschet, and M. Bouillaud has of late years introduced the thermometer into his clinical practice. He has also laid down some general principles derived from the observation of 300 cases, but he has not followed the subject in any of its details. M. Donné has studied the modifications of animal heat produced by several diseases, as well as their relation to the pulse and respiration; M. Piorry, too, has published some curious observations, and M. Andral has deduced several general laws from the various facts collected by himself and others. What these observers have done for the adult, M. Roger proposes to effect for the child; and, for this purpose, he has devoted several months to the assiduous observation of the numerous cases the large Children's Hospitals of Paris presented to his notice.

1. *Mode of appreciating the Temperature.*

The indications furnished to the hand are too uncertain to be of avail, so that a *Thermometer* must always be employed. This should be a delicate one, having a scale sufficiently divided to admit of the 4th or 5th of a degree being distinguished. In the experiments to be adverted to, it has always been placed in the axilla, with the walls of which it can, in the child, be kept completely in contact by bringing the arm across the chest. If the examination is made in Winter, the bulb should be slightly warmed in the hand prior to its application, for if too cold it will startle the child and render him restless. With such precaution we may often pass the instrument into the axilla of young infants without even awakening them. Ordinarily the quicksilver attains its height within three minutes, but M. Roger leaves it in the axilla for five minutes, during which time he also counts the pulse and the respirations. In children, who, from their age or their indocility, may be expected to become restless, it is better to commence by noting these phenomena; and in very young infants, clothed up to the neck, the respirations are best counted by observing the dilatations of the alæ nasi, and when, in such subjects, neither the radial or the temporal pulse can be felt, we must employ the stethoscope. The thermometer should be read off *in situ,* for, if it is as delicate as it ought to be, the quicksilver will descend at least a degree during the few seconds occupied in its removal. When it is desired to take the temperature of the mouth, we are recommended to place the thermometer under the tongue, to prevent the cooling it would undergo from the air passing through the cavity if placed on the dorsum. In tractable children and very young infants this is practicable, the latter, mistaking the bulb for the nipple, close the mouth willingly upon it, and when they open it to cry upon discovering their mistake, we must close the jaws firmly to prevent the admission of cold air. This is not practicable or safe in turbulent children, or such as have teeth, and in these M. R. recommends the instrument should be passed between the cheek and the closed dental arches.

After declaring that he has considered no conclusion satisfactory without verifying it several times, and denying that the temperature of the surrounding medium (this was never higher than 68° F.* or lower than 54°), has had any influence upon the results, the author proceeds to consider

2. *The Normal Temperature of Children.*

Very erroneous *à priori* conclusions have been formed upon this subject. Thus, some authors, observing the activity of function in the child, and especially the rapidity of its pulse and respiration, have concluded that its temperature must be much higher than that of the adult; while others, reasoning from the naked condition and feebleness of the infant, declare it must be much lower. Even experiments differ in the results they furnish. Thus Liebig, without however indicating the experiments whence he has

* To avoid repetition of this initial, we may observe we have, throughout the article, reduced the numbers of the Centigrade scale to those of Fahrenheit's, in doing which we have found Mr. Taylor's "Thermometrical Table" a valuable aid.

deduced so high a figure, places it at 102°; while Edwards found the average temperature of ten healthy infants, from some hours to two days old, to be but 94½°. In three children, from one to two days old, M. Despretz found the average to be 95°. M. Roger believes that, if we wish to arrive at exact results, and reconcile the discrepancies of authors, we must place children in three separate categories.

A. *During Birth* the child has a temperature equal to that which it possesses some days or even some years later. The mean of two examinations, made immediately after the expulsion of the fœtus, gave 99°; and in 8 out of 11 cases the temperature was found two or three degrees higher in the child than at the axilla of the mother. The high temperature of the child during birth may be explained by that of the uterus which it has just quitted, although some observers have certainly placed this too high. Granville states it be 106 in normal labour and 104° after delivery. M. Gavarret found the thermometer to be at 104° during parturition in the sheep. In three or four minutes *after delivery*, the child's temperature sinks to 97°, and then to 95°. Such diminution is probably due to the temporary exposure of the damp surface of the child to the air, and the refrigeration would probably equal that of young animals removed from their parents, if the infant was deprived of the protective care its helpless condition calls for. It is not permanent, the thermometer mounting up again after the next day, so that the mean temperature of five children one day old was 98½°. Although, therefore, in passing into a medium 30 or 40 degrees colder than that which it has left, the child easily parts with heat, its loss is only temporary, and Edwards has stated the medium temperature of new-born children too low (94½). Twenty children, in good health, and aged from one minute to two days, furnished M. Roger with a mean of 98°. In nine of these experiments, in children from 1 to 30 minutes old, the temperature seemed to be independent of the circulation; for the maximum (130) and the minimum (96) of pulsations correspond with the same degree of temperature (96°). Nor is there a greater correspondence observed between the maxima of temperature and of the respirations. Nevertheless, the same child furnished the three minima, viz. pulse 65; resp. 22, and temp. 95½°.

B. *New-born Children.*—On analyzing the temperatures of 33 children, aged from 1 to 7 days, 98½ was found to be the most frequent figure (14 times), 8 children offering a lower and 11 a higher temperature. The mean, however, is 98½, the maximum, observed only once, being 102°, and the minimum, observed also but once, 96½°. The rapidity of the *respirations* seems to have borne but little relation to the temp. which was the same in those in which they were less, and those in which they were more than 36 per minute. The mean number was 39, the minimum 24, and the maximum 86. This last high number occurred in a child 2 days old, who was lying quite tranquil with a pulse only at 84, and a temp. of 98°. The relations of the *pulse* and the temp. seem to have been more constant. The maximum pulse was 140°, the minimum 70, and the mean 102. Fifteen children who attained the mean, or were below it, had a mean temp. only of 98°, while in 18, who exceeded the mean, the temp.

was 99°. *Strong* infants gave a mean of 99½, and *feeble* ones but 98. The mean temperature of 13 boys exceeded that of 16 girls by about a degree. It has been usually said that the temperature is lower during *sleep*, but in 14 infants examined, M. Roger found it about half a degree higher.

c. *Temperature in Early and Advanced Childhood.*—The mean temp. of 25 children, aged from 4 m. to 14 ys. was found to be 99°: being slightly lower for those who were less, and slightly higher for those who were more than 6 years old. The *pulse* in the former group averaged 102, as in new-born children, and in the latter group but 77—shewing that the calorific function cannot exclusively depend upon the circulation, inasmuch as with a slower pulse we here have a higher temp. The *respirations* present a mean of only 30, instead of 39, in the new-born child; both these and the pulse are liable to fewer oscillations, and are separated by less considerable extremes than at an earlier age. The minimum pulse of the 25 children was 64, and the max. 120, a difference of 50 instead of 70. The minimum of the respirations was 20 and the max. 44, a difference of 24 instead of 42. Rapid *exercise* sometimes raised the temp. a degree, but not in other cases, although the number of the p. and resp. were of course augmented. M. Roger's experiments do not confirm the opinion that the temp. increases after a repast.

3. *The Comparative Temperature of different parts of the Body.*

As the result of experiments made upon 15 children, of from 8 to 13 years of age, the various parts of the body are thus ranged according to the greater height of their temperature: the axilla, the abdomen, the mouth, bend of the arm, the hands, and the feet. The axilla and abdomen, in some cases, gave the same temp., but, as in disease, that of the latter is often found lower, while the thermometer is more conveniently applied to the former. In 5 out of 6 experiments the temp. of the mouth was lower than that of the axilla from ½ to 7°. The temp. was usually 2° lower at the bend of the arm, that of the hands from 2° to 11° lower, and that of the soles of the feet from 10° to 13° lower. These data prove there is no equilibrium maintained over all parts of the body, and that the animal heat diminishes in proportion as the centre is receded from. It is highly important to bear in mind such normal differences, when considering the results observed during the presence of disease.

4. *Temperature in the Diseases of Children.*

Although the physiological actions of the economy do not influence the temp. to any considerable extent, either in the adult or child, it is very different as regards disease. Andral, speaking of adults, says that the limits of the variations produced by pathological causes extend over 13°, viz. from 95° to 107½°. In childhood, the limits are far more extensive, as they may be found as low as 72° and as high as 109°, giving an oscillation of 37°; but, like the diseases themselves, exhibiting variations not found at other periods of life.

A. *Diseases in which the Temperature is increased.*—1. *Fever.* Whatever may be the form, type, duration, cause of, or concomitant alterations in fever, an increase of temperature is, as Andral remarks, its characteristic and fundamental phenomenon. He states that, of 350 cases of primary or symptomatic fever, the thermometer stood at 100½° in 64; at 102° in 150; at 104° in 116; at 106° in 18; and at 107½° in a case of acute farcy. To proceed to the varieties of febrile action.

A. *Ephemeral Fever.*—This is seen oftener in children than in adults, and more frequently in private than in hospital practice. Two cases only here are noted, the temperature of the one being 105°, and of the other 101°.

B. *Intermittent Fever.*—M. Gavarret has shewn, in a memoir published in the fourth volume of "*L'Experience,*" that there is an augmentation of temperature not only in the hot but in the cold stage of this disease. In six persons, from 18 to 36 years old, in whom he had the opportunity of applying the thermometer, he found the temperature rose from 97½ to 100½°, and in four trials as high as 104°. In five experiments made during the hot stage he found the thermometer stood only 2° higher than in the cold one. Some explanation is, however, required before attributing the sensation of cold to a mere perversion of sensibility. It is in fact due to the unequal distribution of heat; for, although the temperature of the central organs, ascertained at the axilla, is higher, that at the extreme parts of the body, as the nose, feet, hands, &c., is lower than it should be, as M. Gavarret has subsequently stated. It is in fact but an exaggeration of the normal distribution of caloric, and a partial refrigeration at the extremities may occur in ague, just as we see it normally in Winter, and pathologically in diseases of the heart. The children brought to the *Hôpital des Enfans* are by no means favourable subjects. A great number of these have been long left at nurse in the departments where intermittent is endemic, until they have become cachectic, and their spleens enormously enlarged. The intermittent frequently becomes converted into continued fever, or, at all events, its paroxysms are rendered less certain and regular. For these, and other reasons, it is rare to have the opportunity of observing the temperature during the cold stage in the child. From the examination of 15 cases, it was found that there was a slight diminution of heat in the interval of the paroxysm, or some time after the hot stage had ceased. During the hot stage the temperature was found to rise not quite so high, by about two degrees, as in the adult. In five cases, in which they were noted, the pulse and respiration were always found accelerated during the hot stage, and slower during the cold one.

C. *Typhoid Fever.*—This disease is that of all the affections of children which furnishes the most intense and abiding heat, and that which best proves that man must possess, within himself, a source of heat, independently of the circulation and respiration. The trials, 98 in number, were made upon 23 boys, mostly between 9 and 12 years of age; of these, 14 attained the high temperature of 104°—the maximum being 106.° Leaving out some very slight cases, we have 16, the mean of whose united temp. was 104½°; and even when the slight cases were added, the temp.

was still 103½°. The persistence of the high temperature is also remarkable. Thus, in one case, the therm. indicated 104° to the 8th day, 102° to the 11th, and 101° to the 28th day. In another case the therm. was at 104½° to the 10th day, and 6 days after still at 104°. In another it was at 105° on the 11th of June, and at 103° on the 22nd. As a general rule the temperature was found highest in the most severe cases, varying from 104° to 105°; but in some slight ones it was also very high, and indeed the max. temp. 106° was observed on the 16th day of a simple typhoid, the pulse being only 92, and the cure completed in less than a month. In typhoid fever the temp. is increased from the very beginning; for in four children it was at 105° to the 5th day, at 106° on the 6th day, and at 104° on the 7th and 8th days. This early elevation of temp. is usually proportionate to the suddenness with which the disease has appeared: and, in some instances, such increase has been the first phenomenon announcing the existence of typhoid fever—it being higher than other existing symptoms explained. The temp. usually increases or decreases as the disease becomes more or less severe, and it will even vary with the alternations of its progress. Thus, in one case, to the 29th July, the disease was only indicated by the temp. 101°; on the 2d August, it became more evident, and the temp. rose to 104°; the condition of the patient improving, the therm. descended to 103½°; and by the 9th Aug. stood at 102½°. In the *adynamic* form, the temp. may not be high, although danger may be present—the calorification seeming to participate in the general feebleness of the system. A child, on the 15th day of a fever, which proved fatal on the 18th, gave only a temp. of 99°, and never exceeded 101½°. In another case the therm. was at 104° to the 8th day, after which, adynamic symptoms becoming marked, it sank (together with the pulse and respirations) to 102°, then to 101°, and, just prior to death, to 98½°.

The numbers of the *pulse* are not usually in harmony with those of the temperature, and there is *no other disease of childhood in which the great elevation of temperature contrasts thus with so slight an acceleration of pulse.* Thus the high temp. 104°, 105° and 105½°, have been observed in cases wherein the pulse was but 108, 106, or 96. In one case 104° corresponded with only 88, and in a child which presented the high temp. of 106°, the pulse only reached 92, its normal number at that age being 102. If we add together the number of the pulse, and the degrees of heat, in nine cases of severe typhoid, we find the high mean temp. 104½°, corresponding to the low mean pulse 115; and, taking all cases together, the mean 103½° corresponding also with the mean pulse of 115. So also the *respiration* is not markedly accelerated; since the mean was but 37°, the normal number being 30°. The relation of the respiration and temperature is also far from being invariable. Thus, the max. respiration (60°) corresponded only with 102° or 103°; while the max. of heat (105½° and 106°) corresponded only with 32 and 36 resp. So, too, in observing cases for several days, we sometimes find the respiration remains the same, while the therm. rises or falls from ½ to 2°, and sometimes becomes accelerated while the temp. lowers, or, what is rarer, becomes slower while that rises. Nevertheless, in most cases, there is a relationship between the two phenomena, and their simultaneous decrease is striking during the decline of the disease.

*Practical Applications.*—The above facts are valuable in aid of the *diagnosis* of the diseases of children, sometimes so difficult. Thus, as there is no other disease having so high a temperature with a pulse so little accelerated, if we observe a case in which with the pulse at about 100 the therm. indicates 104° or 106° we may almost certainly pronounce it one of typhoid fever. In some cases a very high temp. should lead us to expect typhoid, although no other symptom be present, while, in others, a moderately high temperature is a corroborative sign. In distinguishing typhoid from *enteritis,* which is not so easy as some imagine, as also ataxic typhoid from *meningitis,* the recollection of the higher temperature in typhoid than in either of these two diseases, especially the former, is of great service. As to the *therapeutical* applications, we see from the enormous and long-continued generation of caloric in typhoid, the explanation of the great utility of the employment of water, both externally and internally, in the form of tepid baths, cold applications, lavements, &c. "How many times, in the wards of MM. Guersant, Rayer, and Recamier, have I had the opportunity of observing the happy effects of the repeated administration of tepid baths and cold drinks in typhoid fever. In more than one case a unhoped-for cure has followed the use of cold irrigations."

D. *Variola and Varioloid.*—The trials made in 9 cases of variola furnished the maximum of 106°, the minimum 99° and the mean 101½°. The mean, however, undergoes variations, according to the stage of eruption, viz. 1st day 106°; 3rd 99°; 5th 102°; 6th 101½°; 7th 105°; 8th 101°; 9th 102½; shewing that the temp. is at its max. at the commencement of the eruption, sinking on the following days until the 5th, when it rises again, this being the period of the suppurative fever. The intensity of the eruption does not influence the temp. to the extent that might be expected: thus the mean of three confluent cases was 101½°, and of 5 discrete and varioloid 101°. The mean here stated is somewhat less than that given by Andral (102°) from his observations on 15 adults. This may arise from the fact of 3 out of the 12 children observed being examples only of varioloid. Although the maxima of the respiration and pulse do not always correspond (the pulse of the child who had a temp. of 106° was but 132, while in the child who had the max. pulse 152, the temp. was only 102°), yet in all the 5 cases in which the temp. was highest, the pulse was rapid (mean 135), and in all those, with one exception, in which the pulse was rapid the temp. was high. The correspondence prevails more strictly still as regards the minima. The relation of the *respiration* was almost always constant in the maxima, but frequently not so in the minima.

E. *Scarlatina.*—Currie has noted the temp. 108°, 109°, and 110°, but M. Roger believes some error in regard to these numbers. He found, in experimenting on 7 children (as Andral also had noted in 7 adults), that the maximum was 105½, the minimum 100½, and the mean 103°. As in small-pox, the high temp. is remarkably persistent throughout the course of the disease. The mean of 14 experiments, made at different periods, was 102½, *i. e.* nearly as high as at the commencement. The increase is proportionate to the intensity of the eruption, the gravity of the symptoms, and the complications of the disease. Those who died furnished a mean of 104°; those who recovered of 101½°. There is, in general, a want of accord between the degree of temp. and the *pulse*. In two cases in which the pulse was 164 the temp. was but 103°, while the max. temp. 105½ corresponded with a pulse of 140. The minimum pulse 108 corresponded with the temp. 103½, and the minimum temp. with a pulse of 125. In general, the pulse was much accelerated, giving a mean of 135. The relation of the *respiration* is more exact, but not constant. The maximum respiration (50) corresponded with the highest temp., but the minimum resp. corresponded with the high temp. 103½. The mean was 37.

F. *Measles.*—From experiments made on 18 patients, the mean was found to be less than in the two other exanthemata, viz. 101°; the maximum being 104° and the minimum 99½. In 11 adults Andral found the temp. was nine times between 100½ and 102, and twice 104°. The duration of the high temp. is not so long as in the other febrile affections. In all, except two cases (in which bronchitis and pneumonia were present), it gradually declined: thus the mean on the 1st day was 102¼; on the 2nd 101¼°; on the 3rd, 101°; on the 4th 99½; on the 5th 98½; and the mean of 30 experiments made at different parts of the course of the disease was only 100°. The *pulse* is more absolutely increased than the temp; and in the majority of cases there is an exact correspondence with their relative increase. The diminution of the pulse and temp. is also simultaneous. The relation of the *respirations* is neither so frequent or so exact. The child who had the max. of respirations (64) furnished only the temp. 102°, while in another with a temp. of 104° the respirations were only 32. Nevertheless, in the majority of cases, the resp. was most accelerated in those cases in which the temp. was highest.

G. *Erysipelas.*—Only two cases were noted. In one, æt 6, the therm. stood at 103½, and in the other (æt. 13) at 104½°.

2. *Affections of the Heart.*—One case of pericarditis and one of hypertrophy, were the only ones met with, and in neither was the temp. much raised, varying from 98½ to 100½. Andral has also shown the slight elevation which took place in the adult, viz. 98½ at the axilla, while applied to the hands the therm. stood only at 86°, and to the feet at 80½. In one case, M. Donnè found the temp. to be but 93°, and in 11 others, it oscillated between 98½ and 100½°.

3. *Affections of the Digestive Organs.*—A. *Stomatitis.*—Although the normal temp. of the mouth is lower than that of the axilla, it is in this affection increased from ½ to 1°. Of four cases the max. was 101½°, the mean being in the mouth 100½, and in the axilla 99½.

B. *Muguet.*—The maximum of 7 cases gave 102°, the minimum 98½, the mean in the axilla being 99½°.

C. *Enteritis* gives rise only to a slight increase of heat; for of 8 boys, from 3 to 13 years old, upon whom 15 experiments were performed, the max. obtained was only 102½, the mean being 100½. The temperature is usually higher in proportion to the acuteness of the symptoms. The minima of the pulse and respirations are oftener in accordance with those of the temperature than the maxima.

The thermometer, as already observed, may be made very useful in distinguishing *typhoid fever* from *enteritis* at an early stage. Compare the high temp. of typhoid 104° with the moderate one of enteritis (100½)—the pulse being of the same velocity in both. In the first, too, the great heat once produced, may continue for weeks, while in the other it disappears in a few days. In many of these difficult cases of *typhoid enteritis*, for the detection of which Rilliet and Barthez declare our means are insufficient, the thermometer will often decide our doubts. If the temp. of 100½ or 100° is maintained for some days, and never rises beyond 102°, we have to do with an enteritis; while, if the therm. rises to 106° or even 107°, the disease is undoubtedly typhoid fever.

D. *Dysentery.*—Three fatal cases of this disease were observed, in which the temp. varied from 100½ to 101°. The thermometer, applied to the abdomen, opposite to where the worst lesions were found after death, denoted from ½ to 1½° lower temp. than in the axilla. The temperature usually continued the same throughout the whole course of the disease: thus, in one case it was 101° on the 26th Sept. and continued the same 16 days after. The same temperature was observed notwithstanding that the symptoms and lesions were very different; and in general there was no connection between it and the frequency of pulse and respiration.

E. *Acute Peritonitis.*—The mean of nine experiments made in three cases was 103°, the therm. standing nearly at the same degree for 5 or even 10 days. The relation to the pulse and respiration existed in one case and not in another.

4. *The Nervous System.*—A. *Meningitis.*—48 trials were made upon 6 cases of cerebral meningitis, and 3 of cerebo-spinal (from 10 months to 14 years of age), and comparing them with other affections, we are struck by the *great inconstancy of the results.* Thus, in typhoid fever, pneumonia, &c. in the great majority of cases the temp. is always high, as it is, but less so, in enteritis, measles, bronchitis, &c.; but in *meningitis* the greatest varieties are met with. The elevation may be moderate in some cases, in others higher than in any other disease whatever (109°), and in others, again, lower than in any other acute inflammation (95°). By an examination of the table given it is found that not one of the maxima exactly resembles another, half a degree at least separating them. The increase of temp., however, oscillates between 103 and 101°, the mean of the maxima of the 9 children 102°, and of 48 trials 100½°. In most diseases attended by increase of temp. this is usually found at the commencement of the disease, and sometimes at the middle, but in meningitis it is oftenest observed towards the end; so that, comparing the trials made at an early and at a late stage of the affection, a difference of 7° is observed. The highest temp. (109°) was noted only an hour before death, and an hour after death the therm. still stood at 102°. Sometimes the rise is sudden and inexplicable; and thus in one case it was 99° on one day and 109° the next, without any additional bad symptoms. Neither the age of the patient, the period, seat, or gravity of the disease, or inflammatory alterations, explain the reason of these differences, as they have been met with under exactly similar circumstances in these respects. The more rapid the *progress* of the disease the higher was the temp. observed; so that in three cases, whose mean duration was 4 days, the temp. was 105½, but in others, giving a mean duration of 21 days, it was but 100½. The relations of the *pulse* do not explain these irregularities; for, although in no other acute phlegmasia do the temp. and pulse descend so low, the following table exhibits a want of correspondence in such descent.

| | | | | | |
|---|---|---|---|---|---|
| "Maximum | Temperature | 109° | Corresponding | Pulse | 160 |
| „ | Pulse | 164 | „ | Temp. | 106° |
| Minimum | Temperature | 95° | „ | Pulse | 120 |
| „ | Pulse | 48 | „ | Temp. | 98½°" |

Thus, although the low number of pulse ordinarily corresponds with a low temperature, reciprocity does not prevail, since we find the great diminution 95° corresponding with a pulse of 120. The correspondence of the *respiration* is better marked; and the pulse, resp. and temp. are more frequently in accordance than not so.

For the purposes of *diagnosis* it is important to remark, that a considerable abatement of temp. may take place at the middle period of this affection, *meningitis being the only inflammatory or febrile disease in which this is the case.* Thus, in one case, the therm. descended from 98½ to 97½, and in another to 96°. It is familiarly known that the *pulse* may undergo similar changes, and that, rapid at the commencement and termination of the disease, it may become slower in the middle period. In one case it fell from 120 to 52, while in another it rose, after being 4 days at 48 and 58, to 140. So the *respirations* have fallen to 14 or even 12.

"Thus, if in the course of a disease presenting cerebral symptoms, the therm. indicates 95° or 97° after having stood at a higher point some days before, we may almost positively declare a meningitis is present, and our certainty will be the greater if there is a coincident abating of the pulse and respiration. So that we may state that a diminution of heat intermediate between two periods of augmentation is a pathognomic sign of meningitis." "In other cases we may distinguish the meningitis from typhoid fever, inasmuch as the mean is 102° for the former and 104½ for the latter: so that, in doubtful cases, in which cerebral symptoms predominate, if the temp. surpasses 106° typhoid may be presumed to exist, while, if it is below 102°, the disease is probably meningitis. But here we cannot speak with the same certainty as in other diseases."

B. *Disease of the Brain.*—In four cases of different descriptions of disease of the brain the increase of temp. was but slight, the maxima being 102 and 103°, and the minima 100 and 101°. The mean of the four cases, although two had a coincident pneumonia, was 101½, and of the 14 experiments made upon them 99°. The *pulse*, on the contrary, was rapid, giving so high a mean as 143, and the *respiration* was accelerated, but neither in proportion to the temp. In affections of the substance of the brain there is not the sudden intermediate lowering of temp. pulse and respirations observed in meningitis.

5. *Respiratory Organs.*—A. *Croup.* From 20 trials made in three cases, it appears that there is no great increase of heat (mean 100½), and by no means to the extent to have been anticipated from the mean rapidity of pulse (152) and respirations (51).

B. *Bronchitis,* when unconnected with pneumonia, gives rise to a much less high temp. than that disease: only once out of 12 cases and 21 trials, did it reach 102°. Taking only the severe cases we have a mean of 101°.

"The distinction between *capillary bronchitis* and *pneumonia* is often made with great difficulty, and if we find a child with fever, cough, dyspnœa, and sub-crepitant *râle* on both sides of the chest, we may have some difficulty in offering an exact opinion. The thermometer may aid us. If it denote a temp. of 104° or 106° there is almost certainly pneumonia, while if this do not exceed 101° there is most probably but bronchitis. The importance of this observation was shewn in the first case of the table. The child had a pulse of 152 (afterwards 192) and 58 respirations, while auscultation revealed small crepitation over the whole chest. Although there was no thoracic dulness, as this sign is wanting at the commencement of diffused lobular pneumonia, I concluded from the intensity of the symptoms that this disease was present. But the autopsy, by exhibiting the integrity of the pulmonary tissue, and the changes in the capillary bronchi, proved to me that I should have avoided this error in diagnosis had I trusted to the thermometer, which only indicated 100½."

*The pulse and respirations* are always very rapid (the mean of 3 cases gave r. 64, p. 132, although the temp. was but 99°) compared with the moderate increase of heat; but the correspondence between these is not always observed.

C. *Pleurisy.*—The temp. in acute cases of from 4 to 6 days' duration was found to be 102°; and in chronic cases, of from 7 days' to several weeks' duration, but 99°. Andral gives the mean in adults at 102°, but Donné, from four trials, places it at but 100½. The relations of the *pulse* and *respiration* are pretty constant.

D. *Pneumonia.*—Of all the diseases of the respiratory organs this furnishes the highest temperature. In ⅔ of 17 cases the therm. surpassed 104°, and the mean of the whole was also 104°, all but a fraction. As in typhoid, the heat here is remarkable for its persistence; so that the 47 trials, made in various parts of its course, still furnish a mean of 102½. It is, however, at the onset and middle portion of the disease that the therm. is very high, becoming depressed towards the end, even when the disease is to terminate fatally. It stood at 106° on the 27th March, and at 101° on the 31st. This descent is not constant or regular, and does not even in the end amount to much, inasmuch as the mean of three trials on the day of death gave 102½. It is constant and regular, however, when the case is about to terminate favourably. The seat, form, gravity, &c. of the disease do not produce proportionate elevations of temperature, and uncomplicated pneumonia is attended by a higher temp. than that which possesses complications, although these may be such as are usually attended by increased heat. The agreement of the thermometrical results, as observed in the child and in the adult, is very surprising, when we consider the difference in the nature and progress of the disease at the different periods of life. In 73 cases, Andral found the temp. twice at 100½; 28 times at 102; 36 times at 104; and 7 times at 106°. His maximum and minimum were identical with those observed by Dr. Roger in children, and his mean nearly so (103½).

Of all the phlegmasiæ, pneumonia is that which is attended with the highest temp. and proportionate exaltation of pulse and respiration. In nearly one-half the cases the *pulse* reached 140 or upwards, and in three 160—the whole mean being 133. In 12 trials out of the 47, the *respirations* amounted to 60 and upwards, the maxima being 84, 88 and 96.

"To judge of the relative exaltation of these functions we may state the means, viz. Temp. 104°, Pulse 133, Resp. 52. These amounts are surpassed by those of no other disease, and in no other is the agreement of the three so well marked; the pulse and resp. rising and falling with the therm. Such agreement it is important to notice in reference to the differential *diagnosis* of *lobular pneumonia* and *bronchitis*. At an early period, the most experienced practitioner may confound these, but there is a characteristic which distinguishes them. A temp. of 104° is a common one in pneumonia, while it is never met with in the bronchitis of infants or adults, 102° being the maximum in that disease as it is the minimum in pneumonia. Thus, if in a child presenting the symptoms of either disease we find the therm. to stand at 100°, we pronounce it a bronchitis, while, if it mounts up to 104°, the parenchyma is involved. It has more than once happened to us to recognize a pneumonia by observation only of the dyspnœa and increased temperature."

Before proceeding to the other divisions of the subject, it may be of advantage to exhibit a tabular view of the *mean temperatures* of the principal diseases already noticed.

| | | | |
|---|---|---|---|
| Typhoid Fever . . . | 104½° | Meningitis . . . . | 102° |
| Variola . . . . . . | 101½ | Bronchitis . . . . | 100 |
| Scarlatina. . . . . | 103 | ——— severe . . | 101 |
| Rubeola . . . . . | 101 | Chronic Pleuritis . . | 99 |
| Enteritis . . . . . | 100½ | Acute Pleuritis . . . | 102 |
| Peritonitis . . . . | 103 | Pneumonia . . . . | 104 |

B. *Diseases in which the Temperature is Stationary.*—These affections, consisting of the Neuroses and certain Diatheses or Cachexiæ, may be separated into two categories, viz. those in which the temp. may, under certain circumstances, be *secondarily* increased, as Pertussis and Tubercle, and those in which it remains *invariable*, unless complications bearing no relation to the primary disease occur, as Chorea, Rickets, &c.

1. *Tubercle.*—(Phthisis Pulmonalis and Peritoneal Tubercles.) If tubercles give rise to an increased degree of heat it is only *secondarily*, from the irritation they excite in the tissues in which they are deposited. When this is not present, or has become chronic, the therm. hardly rises above the normal level. The maximum in 12 cases, in which the course of the disease was slow, was 100°. M. Andral also has observed the temp. to be normal in adults when no fever is present. Of 21 patients, 2 were without fever, and the therm. stood at 98½, while in the 19 others, it stood at 100½ three times, at 102° four times, and at 104 three times—giving a mean of 101°. Of 42 experiments by M. Donné, the max. was 103, the min. 96, and the mean 100°. M. Donné found a relation between the *pulse*

and temp. only in 16 out of 42 cases, the temp. being sometimes found low with a rapid pulse, and the contrary. In the children the relation held good in $\frac{2}{3}$ of the cases, and generally as regards the respiration.

In *Cerebral Tubercle*, unless there is attendant inflammation, the temp. is little raised above the normal: 13 trials made in 6 cases furnishing a max. of $100\frac{1}{2}$, a min. of 98, and a mean of 99°.

2. *Hooping-cough.*—Usually there is no variation of temp. unless bronchitis or pneumonia is present. Thus, 7 cases gave a mean of 101° only, although bronchitis and fever were intense in three, and another died with dilated bronchi. The heat though moderate was persistent, for the mean of 16 trials made at different periods was $100\frac{1}{2}$. The *pulse* (mean 128) and respiration (40) were generally accelerated, but their relation to the temp. as often absent as present.

3. *Chorea.*—In 5 cases (æt. from 8 to 13) the max. temp. was 99, the minim. $97\frac{1}{4}$, and the mean 98°. This does not confirm Becquerel and Breschet's statement of the increase of temp. during muscular contraction, and indeed, in a case in which the contractions were constant, it was less than $98\frac{1}{2}$. The pulse and respiration were usually within physiological limits.

4. *Various Forms of Dropsy.*—When dropsy is acute and accompanied with fever, there is an increased temp.; but when it is apyretic the mercury is not influenced, or even slightly depressed. This observation applies to dropsy, whether consecutive to scarlatina or connected with anemia.

5. *Rickets.*—In pale, ill-developed children, whose contracted chest gives rise to imperfect hæmatosis, we naturally expect to find a diminished temp., yet in the cases examined this has not proved to exist, the therm. ranging from $97\frac{1}{4}$ to 100°. If phlegmasiæ attack these subjects, however, the temp. seldom reaches the same height as in healthy children.

6. *Paralysis.*—In the physiological condition we find a difference of 13° between the axilla and sole of the foot, but in a paralytic boy Dr. Roger found the difference to extend to 31° (*i. e.* 68 and 99°); but in other instances he has been as unsuccessful as Andral in detecting diminutions of temp. in the paralyzed part. However it may be in some cases as regards *partial* modification of temp. there is no *general* loss; for in seven experiments the mercury neither rose or fell at the axilla.

c. *Diseases in which the Temperature is diminished.*—When the child has passed through the first few hours after birth, a period at which its calorific powers are at a minimum, no physiological condition gives rise to a diminution of temperature; but under the influence of disease the thermometer may sink many more degrees than it rises in those diseases which are attended with an increase of temperature. The number of diseases, however, in which there is *diminution* of temp. are very few in number; viz. one in which this is *partial*, gangrene of the mouth; and two in which it is *general*—cholera and the œdema of new-born children.

1. *Gangrene of the Mouth.*—The diminution of temp. which follows the application of the therm. to the eschar is far less than that which is present in sphacelus of the limbs in the adult. Dr. Roger observed the case of a man æt. 30, suffering from gangrene of the foot, and found the temp. of the part (70½°) to be less than that of the axilla (100°) by 30°, and only 6° more than that of the circumambient air (64½°); the pulse being 112 and the resp. 30. Eight days after, when the condition of the patient had improved, the temp. had risen to 74°. But in a child with gangrenous stomatitis the temp. was 81, or 19° above the external air (62°), and only 9° less than the healthy cheek (90°.). At the commencement of the affection, when the gangrene is circumscribed, and the eschar thin and not visible externally, the temp. is even raised a little above the normal mean. So, too, the temp. of the mouth, which is normally several degrees less than that of the axilla, was found, in consequence of the surrounding excitement, to be identical with it in three cases, and 1° above it in another. The general temp. is also raised, the pyrexia which precedes the gangrene not being extinguished or diminished in consequence of the establishment of this morbid process. Thus its mean in 7 cases was 102°, the maximum of 104° being once attained. This augmented temp. too, is very persistent, for the mean of 14 trials at various periods gave 100½. The relation of the *pulse*, but especially of the *respiration*, with the general temp. is usually maintained.

2. *Cholera.*—There were but two cases of *sporadic cholera* observed at the *Hôpital.* In one, the therm. at the axilla gave 99°, although the upper extremities and the face were cold to the hand. In the other, the temp. was 97½; but at the bend of the elbow, which is usually but 2° lower than at the axilla, the therm. stood at 90°. These cases do not prove that there is any general cooling in cholera, but rather that it is partial. Yet this result is sufficient to distinguish cholera from other grave conditions (as cold stage of intermittent, peritonitis from perforation), in which, although the surface may be as cold, there is augmented heat at the axilla, and which are characterized not by any diminution of temperature, but by its unequal distribution.

Dr. Roger is not aware that any thermometrical experiments were made during the prevalence of the cholera at Paris in 1832; but does not doubt, from the analogy so many of the symptoms present with those of the œdema of new-born children, that a general diminution of temperature would have been found. Experiments made in Germany, indeed, confirm this opinion. Caspar of Berlin found the temperature in severe cases to be but 79°. Czermak of Vienna found the maximum of refrigeration at the feet to be 64°, at the tongue 66°—the temperature of the blood oscillating between 77 and 91°. Although these experiments are but imperfectly detailed, and may be to some extent inaccurate, they are sufficient to warrant the conclusions of their reporters, MM. Gerardin and Gaimard, that there is no other disease in which the temperature descends as in the Indian Cholera.

### 3. *Œdema of New-born Children.*

This affection, known also under the names of *Induration* of the *Cellular Tissue, Scleremis,* the *Skin-bound Disease,* &c., is attended by a *general* diminution of the temperature; and the thermometer, placed in the situation where heat is usually best preserved, descends many degrees, so as to indicate a lower temperature than that which is found even in children who have been dead for 8, 12, or even 15 hours. M. Roger bases his elaborate description of this singular disease (which is infinitely more common in France than in this country) upon 100 trials made upon 29 cases; and, in all these, the thermometer left in the axilla for five minutes has, *without exception,* indicated a lower temperature than the normal one (98½) of children from 1 to 7 days old. In 19 of the cases the temperature was lower than 91½°, and in 7, lower than 79°—the mean of 52 trials giving 88°, *i. e.* more than 10° less than the normal mean. In extreme cases the mercury sank to 77, to 74, to 72, and even to 71°, or 27° below the ordinary temperature. The cooling is also remarkable from its persistence, in spite of contrary influences, nothing being able to oppose it, not even the presence of plegmasiæ, which otherwise augment the temperature. In more than half the cases there is developed a consecutive change in the pulmonary tissue, described by the best writers as *pneumonia;* but as the mean temp. of that disease is 104°, how is it that, in scleremis, whether one or both lungs are inflamed, the thermometer nevertheless continues to descend? "A singular pneumonia, indeed, in which the thermometer falls to 72°, instead of rising to 106°, in which the pulse is at 60, and the respiration as 14. Such a contrast induces us to declare it rather to be the congestion from œdema, which also affects other organs, as the alimentary canal, brain, and liver, than the hepatization of pneumonia."

The low temperature exists from the beginning, and is often the first phenomenon remarked. Thus, in a case of a child five days old, apparently in perfect health, except that a slight œdema of the hands and ankles had been observed for a few hours, the temperature was found at 90°, but numerous trials have failed to prove whether the œdema and low temperature are simultaneous, or that the one precedes the other. The degree of diminution is proportionate to that of the induration, and increases from day to day; so that, when we find the thermometer sinking, a speedy death may be pronounced as certain, while its stationary, and still more rising, conditions, hold out hopes of recovery.

When we consider that a diminution of 3° or 4° is an exceptional phenomenon in health or disease, we cannot but wonder that life should be supported so long under the loss of so many degrees. Most of the children, nevertheless, although usually the delicate and premature are those attacked, continue to exist for 4 or 6 days, or even 8, 10, or 13 days—the temperature of these last never however being so very low. What is the lowest temperature the child may arrive at and recover? In one of the two cures recorded by M. R. it was 90°, and in the other 91°. The system long resists even the greatest diminutions; and thus several children lived from 8 to 12 hours, having a temp. of 74 or 75°, and the one who attained the lowest limit (72°) lasted for almost a day afterwards.

It is a fact, showing that the laws of temperature are very similar in all warm-blooded animals, that these low figures in indurated children are not very far removed from those observed by Edwards, in his experiments upon mammiferæ and birds. New-born animals, isolated from their parents for an hour or two, in an atmosphere of from 50 to 68°, rapidly lost their heat, the therm. often descending to 64°, the minimum being 57°, and once 55°. Chossat, examining the temperature of animals dying from inanition, found the mean to be 75°, and the minimum 64°.

In no other disease does the temperature correspond more exactly with the pulse and respiration. The *pulse* exceeded 100 only in 15 out of 40 trials, and 80 twelve times. It was less than 80 thirteen times, and in two as low as 60. The few times it exceeded the physiological mean (102) the œdema was very slight, or some intercurrent phlegmasia occurred. The *respirations*, too, only exceeded the normal mean 13 times in 39 cases: 26 times they were less, and in six instances fell as low as 20, 16, or even 14.

"The *nature* of this œdema has given rise to many hypotheses. According to Underwood, it is a spasm of the skin, dependent upon intestinal irritation. M. Denis regards it as an entero-cellular inflammation; Baron, as symptomatic of some obstacle to the circulation of the blood in the lungs or great vessels. According to Hulme, Dugès, and Irocon, it is a peripneumonia. Others look upon it as proceeding from an original defect, whether in the shape of permanent flaccidity of the lungs or of a non-obliteration of the fœtal apertures. Uzembezius attributes the œdema to a thickening or stasis of the blood; Auvity, toa coagulation and congelation of the fatty juices. Lastly, Valleix, Paletta, and others, lay great stress, among the causes, upon the congenital debility and the action of cold. In reference to these two important points, *congenital debility* and *external cold*, it is to be observed that, almost all indurated infants are premature, and that, at the *Hôpital des Enfans*, the disease is as rare in Summer as it is frequent in Winter. Children born before their natural time have a lower temperature than those at full time, and the newly-born infant has a great tendency to cool, while Currie has shewn that the temperature may be lowered many degrees by the external application of cold. Is it the thermometrical sinking which produces the simultaneous depression of the circulation, respiration, and power of motion, or are these merely coincidently affected? The experiments of Chossat show that, in animals suffering from inanition, the various functions are depressed as the animal becomes cold, and are revived under the influence of the application of artificial warmth. The lethargic condition of hybernating animals, and many actual experiments upon indurated children, also lead to the conclusion that a *diminution of temperature* is the *primary* phenomenon. However this may be, it certainly is the predominant and essential one: for not only is it present when the pulse and respiration are slow, but also when these are normal, or even in excess. Thus, in one case, the pulse was 124, although the thermometer was at 91°. In another, the temp. being 91°, the resp. were 50; and in a third case, the resp. were 32, although the temp. was but 82°. * * * * * * Although the lesion of caloricity may be stated as the essential condition, we are unable to trace the connection of this with the infiltration of the cellular tissue; so that we are, in fact, but shifting the difficulty of the explanation, as the sinking of the calorific power depends upon some other functional or organic change as its cause, whose nature we cannot ascertain as long as we continue so ignorant of the sources of animal heat."

In *treating* this disease, its effects, therefore, can only be attacked. We should at once seek to dissipate the forming œdema, and restore heat to the periphery, by exciting frictions, and the direct application of warmth, whether by means of hot sand, or hot or vapour baths. When the refrigeration is not considerable we may prefer cold affusion, or rubbing the extremities with ice. In the cholera, the sudden cold produced by such applications was succeeded by a salutary re-action and notable increase of temperature. When the temperature has already sank many degrees, it is raised again by the addition of caloric only with great difficulty, never to the normal mean, and speedily lost again as soon as the application is discontinued, in spite of the child being surrounded by non-conducting articles of clothing. It is only then at the commencement of the disease that any such means can be of avail. If the conclusions of comparative physiology are of any value we should also give aliment: for, according to the observations of Chossat, the digestion of food renders the retention of communicated caloric more easy, and effects that which mere warming will not alone.

### D. *General Observations on the Temperature in Disease.*

The slightest derangement of health with accession of fever will cause an increase of temperature that no variations of the physiological phenomena or exposure to a medium of even 104° will induce. So, although under ordinary circumstances, the system of the infant is enabled to resist, to a certain point, the tendency to cool, when affected by scleremis, we have seen the thermometer descend surprisingly. Thermometrical experiments justify the old division of diseases into Pyretic and Apyretic. It is a remarkable fact, that there should be so few diseases attended by an actual diminution of temperature, and it is to be observed that this is attended with a like peculiarity of other functions ; for, just as the pathological circumstances are frequent under which the pulse and respirations are accelerated, are they rare in which these fall below the mean.

In considering the morbid conditions of temperature, we must distinguish such modifications as are *partial* from those which are *general*. Thus, we may have the temperature generally and uniformly augmented, which is the common circumstance. At other times, the increased heat of the internal organs may be noted at the axilla, while the surface and extremities may be colder, as in ague. The heat may also be augmented upon limited portions of the surface, as in eruptions of the face and stomatitis, when, however, it does not exceed that felt in the axilla, which is also increased. A diminution may also be local, as in some cases of paralysis and gangrene of the mouth, the general temperature being stationary, or even increased, if fever is present. At other times the chill is universal, as in scleremis.

Although sometimes the temperature attains its *maximum* only when the disease is at its height, it is ordinarily at the *commencement* (especially in typhoid fever and pneumonia) that it does so. When a *cure* occurs, the high temperature gradually diminishes, but a temperature above the mean remains during convalescence, whatever may have been the feebleness and exhaustion produced by the disease and remedies, and although the patient is really more sensible to cold than in health. The modification which the temperature undergoes on the *approach* of *death* is various ; for if, in some cases, it becomes gradually lowered, especially at the extremities, as the fatal period approaches, in others, it attains its maximum during the last few hours of life, and the highest figure was observed an hour prior to death.

What are the limits of the *oscillation* of *temperatures* in the sick child? Andral has found this to be very restricted in adults, viz. between 95° and 107½° (cholera not being included); but in children the range is much more considerable, viz. from 72 to 109°. This difference does not arise from the thermometric results in the child and adult differing in the same disease, for, on the contrary, the identity of the figures is almost complete, however different the symptoms, forms, and gravity of the affections may be at the two periods. The absence of agreement is entirely due to the low temperature observed in the œdema of infants. The maximum (109°) observed in children is almost identical with that noted by Andral in adults; so that a rise of 10 or 11° above the mean is the extent which the child will support. Even an increase of 6 or 7° is a very considerable one, so that a maximum of 106° or even of 104° is by no means of frequent occurrence. When we consider the dry and burning sensation, the skin of a fever-patient imparts to the hand, we have difficulty in crediting the fact that the thermometer has only risen some 4° or 5°, and that the extreme limit of its elevation is 109: but we cease to feel surprise upon considering that the various physiological acts of life give rise to insignificant variations, and that plunging the body into the hottest medium it will bear only increases the temp. 4 or 5°. On comparing the experiments of M. Roger with those of Delaroche, Berger, and Chossat, on mammifera and birds, and considering the correspondence of the one with the other, we may conclude that man and warm-blooded animals cannot maintain life if their temperature be augmented more than 12° above its normal mean.

In the child, as in the adult, the thermometer may *descend* much lower than it rises—the inferior limit extending nearly thrice as far as the higher one. Thus, while an augmentation of 10 or 11° will cause the death of the child, a loss of between 20 and 30° may be sustained before this takes place. Yet a loss of 8 or 9° seems incompatible with a return to a healthy existence; for no child has ever been observed to be cured in whom the thermometer has sank below 90°. Czermak states, however, as regards adults, that a cure may be obtained in cholera, although the thermometer may have sank to 75°. However this may be in a gradual diminution of temperature, Currie has shewn that healthy individuals cannot expose themselves safely to the lowering of their temperature several degrees, (from 4° to 12° as a maximum.) The lowest limit to which the thermometer may descend is very much the same in all animals of warm blood as in man, maintaining the analogy indicated for the high temperatures.

*Practical Applications.*—" As we have already observed, the thermometer may be of considerable use in the *Diagnosis* of disease. Increase of heat and a rapid pulse are the constituent elements of the febrile condition; but the first of these possesses much more semeiological value than the second: for the pulse is often rapid both in health and disease without corresponding increase of temp., but increase of temp. always leads to a quick pulse—whence it results that a quick pulse alone could not determine the presence of fever, while the exclusive consideration of augmented heat might do so. It is of the more importance to pay attention to the temperature, as in new-born children a state of perfect health may exist in apparently disordered conditions of the pulse and respiration. The normal oscillations of the pulse vary from 80 to 120, or even 140, and the number of respirations may, in quite healthy children, amount to 50 or even 80; so that we must not believe in the existence of febrile action in such cases, unless the temp. reaches more than 100½°; on the other hand, (very rarely however,) the pulse may be so little increased, that we might overlook the existence of fever if we neglected the careful appreciation of the augmented temperature.

"Although we learn from the thermometer that fever exists, it does not indicate its nature, for the same maxima of temperature may be present in typhoid, in eruptive fever, pneumonia, meningitis, &c. The increase of heat considered alone has then little semeiological value, but, compared with other phenomena, it has much. Thus typhoid is the only febrile affection of children in which we have great heat with a moderate pulse; so that, when we see a child exhibiting a temp. of 104 or 106°, the pulse being only 100, we may infallibly, from this non-correspondence, pronounce a dothienenteritis to be present."

The author makes other observations upon the diagnosis of *enteritis, meningitis,* and *pneumonia;* but to quote them would be only to repeat those we have cited under each of these articles. We may extract some of his observations upon the utility of the thermometer in *Prognosis.*

"As a general rule, an excessive increase of heat must be regarded as a bad sign. Except in intermittent fever, which usually terminates favourably, there is almost always great danger when the therm. reaches 106°, such cases usually terminating fatally. Below 106° the prognostic value of the therm. becomes much diminished; for we have seen the same max. 104 or 105° in an ephemeral fever and a fatal pneumonia. In the pyrexiæ, and especially eruptive fever, the cases in which the heat is most developed are usually those presenting most severity. Thus, in scarlatina and variola, in which the temp. is so much more developed than in rubeola, the danger is likewise much more considerable.

"If, during the premonitory symptoms of an *exanthem,* there is excessive intensity of heat, we must fear a too large development of the eruption, and irregular form of the disease, or some complication; and our prognosis must be unfavourable accordingly. So a high temp. (105 or 106°) at the commencement of *typhoid* leads us to fear a long duration and uncertain results. So too in *acute phlegmasiæ,* the amount of heat developed at their commencement is a measure of their gravity. A high temperature ushering in a *pneumonia* or *bronchitis, enteritis* or *peritonitis,* should give rise to alarm. But, when we compare the phlegmasiæ with each other, the gravity of the prognostic is not alike in all. A meningitis may be fatal with the therm. at 99° or even 97½, while the patient may recover from a pneumonia even if it is at 104°.

"In these febrile affections, which are intense from the commencement, the *maxima* of heat almost always show themselves from the commencement, and we cannot draw any favourable deduction from the mere diminution of temp. which may occur later. When this, however, coincides with other signs of amendment, and is gradual and regular, we hope for a cure. In other cases, where the termination is to be fatal, the temp. after losing its temporary elevation is subjected, to the end of the disease, to irregular alternations, and generally without any relation to the greater or less danger the different phases of the affection may present."

In regard to *therapeutical conclusions,* Dr. Roger agrees with Edwards as to the feeble powers of calorification of the new born-child, and the necessity there is for maintaining its temperature by appropriate clothing and even artificial heat if requisite. He exposes the errors of those who would endeavour to harden these little creatures by exposure to cold, and animadverts upon the murderous practice obligatory throughout France (notwithstanding the often-repeated protests of medical men), of carrying children to the Registrar's Office within the first three days of their birth for the purpose of verifying, and with the certain effect of endangering, their existence.

He also insists in the importance of employing the thermometer for the recognition of such cases as require the application of cold and other measures for the reduction of exaggerated temperature; such application being capable of due regulation only by its means.

Dr. Roger concludes his Essay with reviewing the various theories of *Animal Heat*, and states his belief that none of these are exclusively correct.

"We have seen, in various cases, the three principal functions, respiration, caloricity, and circulation, simultaneously exalted or depressed; but where is the point of departure of the exaltation so remarkable in bronchitis, and especially in pneumonia? How do we explain the simultaneous depression observed at the middle stage of meningitis, in cholera, and still more in the œdema of infants? Is it the cold which benumbs every function, or, on the contrary, the languor of the respiratory and circulatory functions which induces refrigeration? The order of the succession of phenomena escapes us: it is a complete circle, whose initial point is nowhere discernible. Thus, relatively to the obscure and complex question of the theory of the development of animal heat, our thermometrical experiments reject all exclusiveness. Is it not, in fact, more rational to suppose that in the animal economy—in which so many chemical combinations and decompositions are in incessant operation—the sources of animal heat may be manifold, as they are in the physical world? May not the digestive, respiratory, nervous, circulatory, and cutaneous functions, each in an indeterminate degree, contribute to the maintenance of animal heat, which other active functions, as secretion, transformation, pulmonary exhalation, tend to abstract—not to notice the operation of external influences. The derangement of the equilibrium of these contrary forces, whose power has not yet been calculated, may lead to, by a mechanism not yet known to science, the heating or cooling of the body in disease."

In concluding our analysis of this valuable and interesting Essay, we must not omit to state, that the particulars of every thermometrical trial is detailed in the tabular form.*

---

* After we had completed the above article, we met with, in the *Philadelphia Med. Exam.* (No. IX. for 1845), a record by Dr. Dowler of New Orleans, of the temperatures of two cases observed by him, which is too extraordinary not to be noticed. The first one was that of a man æt. 30, who died of acute typhoid fever. The temp. varied in the axilla for two hours and a half after death (that of the dead-house being 94°) from 109 to 110¾; in the rectum, it was 111½, and at the epigastrium 109 and 110°. The other case was one of *coup de soleil.* Temp. in axilla 20 minutes *before death* 111°; 15 minutes *after death* 112°; 1 hour after 113°. The body was now stripped and the axilla exposed, but still gave a temp. gradually decreasing from 112 to 109° for 3 hours after death. The exactitude of these figures may be well called into question! The temp. of the air varied from 80° at 6 A. M. to 96° at 4 P. M.; but that of the day prior to the man's death is from its intensity worthy of record. 23d July, 7 A. M. 85°; at 9, 90°; at 10 in the sun 130°; 2 P. M. near the sun 150°, sand in the street 152°; 8 P. M. 89°. Fifteen persons perished from insolation.

